# Rethinking biological resilience of older adults under climate change: an integrative perspective from planetary health and cultural ethics

**DOI:** 10.3389/fphys.2026.1825414

**Published:** 2026-05-18

**Authors:** Chengmeng Zhang, Yilin Liu, Haoyu Suo, Gong Chen

**Affiliations:** 1 Institute of Population Research, Peking University, Beijing, China; 2 Institute of Ageing Studies, Peking University, Beijing, China; 3 Social Development Research Institute, National Development and Reform Commission of China, Beijing, China

**Keywords:** biological resilience, climate adaptation, climate change, healthy aging, older adults

## Abstract

Global climate change is an increasing challenge to healthy aging because extreme heat, air pollution, sleep disruption, and ecological instability can weaken physiological homeostasis in older adults. As aging reduces thermoregulatory, cardiovascular, immune, endocrine, and autonomic reserves, repeated climate stress may lead to delayed recovery and a biological resilience cascade that increases the risk of frailty, cognitive decline, cardiovascular events, hospitalization, and reduced healthspan. This Perspective proposes an integrative framework that situates biological resilience within psychosocial and cultural contexts. We argue that cultural ethics do not directly alter physiological biomarkers, but may shape upstream social and psychological conditions, including stress appraisal, help seeking, community care, adaptive behavior, and recovery after exposure. Drawing on planetary health and cultural ethics, we examine how Zhi Wei Bing, Ren, Yi, and Tian-Ren-He-Yi can inform climate adaptation for older adults. These concepts support preventive action, care for vulnerable groups, fair distribution of resources, resilience literacy, and ecological planning. By linking physiological mechanisms with psychosocial resources and cultural values, this article offers a hypothesis-generating framework for climate-resilient aging and for policies that protect vulnerable older adults in a warming world.

## 1 Introduction: climate change, aging, and the crisis of resilience

Global climate change is increasingly recognized not only as an environmental crisis but as a profound disruptor of human health, particularly for older populations (Patz and Hatch, 2013; Núñez-Rodríguez et al., 2025a). As extreme weather events, such as heatwaves and severe air pollution, become more frequent, they disproportionately threaten the physiological homeostasis and health trajectories of vulnerable older adults (Meade et al., 2020; Qi et al., 2025). The concept of planetary health highlights that human well-being is inextricably linked to the stability of natural systems, making climate change an urgent public health emergency (Prescott et al., 2022).

To survive these environmental stressors, the aging body relies on “biological resilience”—defined as the systemic capacity to regulate, adapt, and recover from external shocks (Li et al., 2022; Cosarderelioglu et al., 2025). However, the natural aging process inherently diminishes physiological reserves, making the maintenance of homeostasis more energy-intensive and less efficient. Current gerontological and climate research has largely focused on isolated pathways, such as the exacerbation of pre-existing chronic diseases, cellular stress, and localized inflammation (Fuller et al., 2022). While these studies are highly valuable, this fragmented focus often overlooks the systemic loss of resilience. When compounded by persistent climate stressors, the aging body faces a fundamental “crisis of resilience,” where even minor environmental fluctuations can trigger a disproportionate and cascading decline in overall health (Klasa et al., 2021; Zhang et al., 2024b).

Addressing this multi-dimensional crisis requires an expanded lens that moves beyond purely biological or economic metrics (Darmon, 2024). This perspective article aims to bridge this theoretical gap by proposing an integrative health resilience framework that unites biological, psychological, and cultural dimensions (Walston et al., 2023). Specifically, we revisit traditional cultural ethics—such as the Confucian principles of Harmony (Tian-Ren-He-Yi, “天人合一” in Chinese) and Benevolence (Ren, “仁” in Chinese)—not merely as abstract philosophical ideals, but as socio-cultural resources that may shape stress perception, social support, and adaptive behavior (Ip, 2025; Sellmann, 2025). We argue that these cultural ethics function as psychosocial “amplifiers” of biological resilience, buffering stress responses and enhancing systemic recovery (Ye et al., 2024). By integrating physiological mechanisms with cultural ethics, this work provides a novel pathway to inform climate adaptation policies that protect and empower older adults in a warming world. This perspective does not suggest that cultural ethics directly change physiological biomarkers. Rather, it treats cultural values as upstream social and psychological conditions. Research in psychoneuroimmunology suggests that social environments can become biologically embedded through immune and inflammatory processes (Miller et al., 2009; Gough, 2024). The immune system may also respond differently to different psychosocial stressors, with chronic interpersonal stress showing distinct associations with inflammation and depression risk (Gough, 2022). In this context, cultural values may influence how people perceive climate risks, whether they seek help, how communities organize care, and whether older adults feel supported during environmental stress (Pruimboom et al., 2024). Through these pathways, cultural ethics may indirectly affect biological resilience by shaping stress appraisal, health behavior, social support, and recovery after exposure.

## 2 Conceptual framework: climate stress and biological resilience in older adults

The World Health Organization identifies climate change as an escalating public health emergency, one that poses a disproportionate threat to aging populations across the globe (Falchetta et al., 2024). This vulnerability represents a profound issue of climate justice; marginalized older adults—often lacking adequate financial resources, robust healthcare access, or protective infrastructure such as air conditioning and urban green spaces—are systematically exposed to higher levels of environmental hazards (Levy and Patz, 2015; Nunes, 2018). However, this macro-level socioeconomic inequality manifests fundamentally at the micro-level as a profound crisis of biological resilience. In the context of a rapidly warming planet, older adults confront a severe “double burden”: the natural, gradual degradation of their physiological systems compounded by the intensifying severity of environmental exposures like extreme heatwaves and severe air pollution (Zhang et al., 2024b). Biological resilience—defined as the systemic capacity to maintain, flexibly adapt, and efficiently restore physiological homeostasis following exposure to external stressors—is therefore the critical determinant of their survival and well-being.

Climate stress could weaken the body’s ability to recover across several physiological systems. Urban heatwaves provide a concrete example, an older adult living alone in a poorly ventilated apartment may be exposed to high indoor temperatures during both the day and night. At first, the body tries to maintain stability through sweating, widening of skin blood vessels, increased heart rate, and changes in fluid balance. In older adults, these responses may be less efficient because of reduced thermoregulatory capacity, lower cardiovascular reserve, chronic disease, or medication use (Lv et al., 2025). If the older adult cannot cool down or obtain help, recovery after heat exposure may be delayed. Repeated nights of poor sleep, mild dehydration, and cardiovascular strain may then increase inflammatory activity, reduce autonomic flexibility, and raise the risk of frailty, cognitive fatigue, or acute cardiovascular events (Ren et al., 2011; Choi et al., 2023). Air pollution may further worsen this process. Prolonged exposure to ambient pollutants can trigger chronic immune activation and low grade inflammation, which may deplete biological reserves and accelerate cardiovascular aging (Kumar et al., 2025; Münzel et al., 2025). Together, heat exposure, air pollution, sleep disruption, and dehydration can impair the aging body’s ability to sense, regulate, and recover from environmental stress, that is often reflected in reduced heart rate variability during extreme weather (Ren et al., 2011). This condition illustrates how climate stress can move from environmental exposure to physiological dysregulation and finally to adverse health outcomes. For this reason, climate adaptation policies should not only respond to visible illness after it occurs, but should also protect the fragile physiological baseline of older adults before stress becomes clinically severe (Zhang and Chen, 2024).

Building on this example, we use the term “Resilience Cascade” to describe the process through which repeated climate stress moves the aging body from temporary compensation to delayed recovery and then to clinical vulnerability. When an individual encounters an acute environmental stressor, the body’s autonomic and endocrine systems immediately attempt to compensate to preserve internal stability (Gideon et al., 2025). In younger or healthier individuals, this adjustment is often short lived, and the body may return to baseline after the stressor ends. However, in older adults, reduced physiological reserve may make this recovery slower and less complete (McKenna et al., 2023). Over time, repeated episodes of incomplete recovery may contribute to higher allostatic load, greater frailty risk, and reduced capacity to withstand future environmental shocks.

Beyond these physiological pathways, understanding resilience in aging populations requires a broader conceptual framework that integrates biological processes with psychosocial and cultural determinants. Biological resilience does not operate in isolation. Rather, it emerges from dynamic interactions between the body’s regulatory systems, the social environments in which individuals live, and the cultural values that shape perceptions of risk, responsibility, and adaptation. For example, empirical studies have shown that social connectedness is associated with lower systemic inflammation and improved cardiovascular regulation in older adults, suggesting a direct link between social environments and physiological resilience (Qi et al., 2023; Granieri et al., 2026). Similarly, perceived control and psychological well-being have been linked to more adaptive stress responses, including improved autonomic regulation and reduced cortisol dysregulation under environmental stress (Meier et al., 2026). At the community level, supportive social networks during extreme climate events, such as heatwaves, have been shown to reduce mortality risks among older populations (Xi et al., 2024). These findings collectively suggest that resilience in aging is shaped by multi-level interactions that extend beyond purely biological mechanisms.

The integrative framework proposed here has three levels: the first level is exposure, including heat, air pollution, poor sleep, and other climate-related stressors; the second level is physiological regulation, including thermoregulation, cardiovascular workload, immune activation, endocrine response, and autonomic control; the third level is social and cultural modulation. At the third level, family support, community care, perceived control, and cultural values may affect whether older adults avoid exposure, receive timely help, and recover after stress. Social connectedness, perceived agency, and community support may buffer physiological stress responses, whereas cultural traditions can reinforce these psychosocial resources by providing shared ethical orientations and collective meaning. It suggests that climate stressors initiate a biological resilience cascade, while psychosocial and cultural mechanisms function as modulators that influence the trajectory of recovery and adaptation.

Within this framework, resilience is not simply a biological attribute but a multi-layered process that links physiological stability, social organization, and cultural worldviews. This structure helps explain why biological resilience in aging is not only a property of the body, but also depends on the social and cultural conditions in which the body responds. By situating biological resilience within broader socio-cultural contexts, this perspective expands current climate-health research beyond disease-centered models and highlights new pathways for strengthening adaptive capacity among older adults in a warming world.

## 3 Cultural roots of health resilience: “Zhi Wei Bing” and “Tian-Ren-He-Yi”

**Figure 1:**
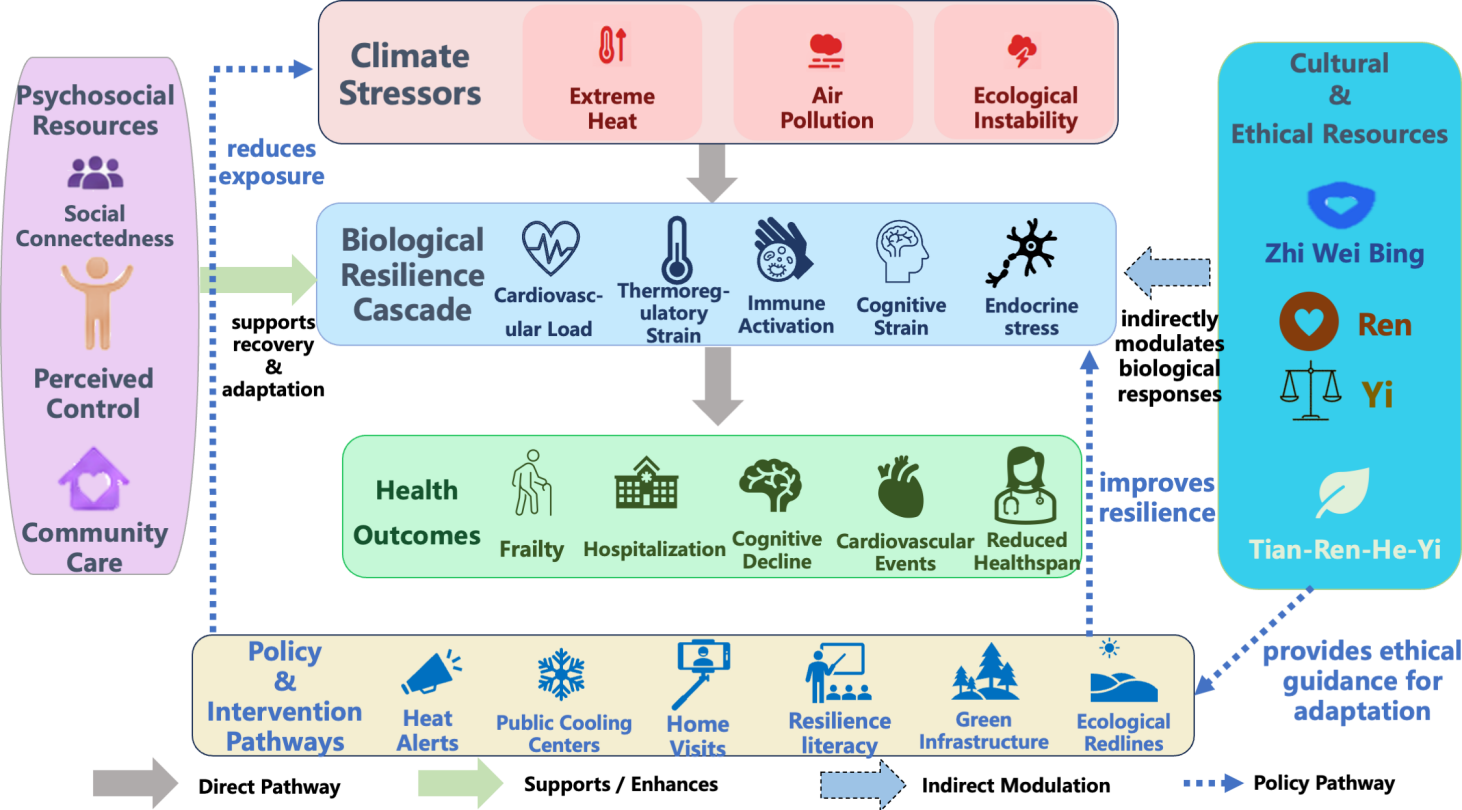
Integrative framework of climate stress, biological resilience, and socio-cultural modulation in older adults.

To effectively interrupt the physiological resilience cascade triggered by climate extremes, adaptation strategies must extend beyond physical infrastructure to encompass profound cultural philosophies. The conceptual framework of this article draws on two related traditional thoughts: the Traditional Chinese Medicine (TCM) concept of “Zhi Wei Bing,” which refers to preventing disease before its onset through early intervention and risk reduction; and the Confucian ideal of “Tian-Ren-He-Yi,” which refers to the unity of humans and nature and is often understood as the need to keep human activity in balance with natural systems (Xu et al., 2025; Zhang and Sargent, 2026). As illustrated in **Figure 1**, this coupled mechanism demonstrates how ancient cultural wisdom translates into modern socio-cultural modifiers, actively buffering both the physiological and psychological impacts of climate stress on older adults.

### 3.1 The TCM concept of “Zhi Wei Bing”

Traditional Chinese Medicine has long championed the concept of “Zhi Wei Bing,” which essentially translates to “treating a disease before it occurs (Lou and Wu, 2025).” In the context of modern planetary health, this preventive orientation aligns perfectly with the enhancement of biological resilience by building the aging body’s adaptive capacity and physiological reserves before pathological damage occurs.

Historically, while weather-related ailments were acknowledged in traditional medicine, severe climate-driven environmental stressors were not as catastrophically prominent. Today, however, as the acute health risks posed by unprecedented micro-climate variations, urban heatwaves, and pollution spikes become increasingly recognized, the necessity of “Zhi Wei Bing” has been brought to the forefront of public health. Rather than passively allowing the resilience cascade to advance toward compensatory failure and clinical frailty, “Zhi Wei Bing” emphasizes action before illness becomes clinically visible (Kwon et al., 2026). In the context of urban heatwave, this approach would start before heat illness appears. It needs to identify high-risk older adults before the hot season begins first, especially those living alone, those with cardiovascular disease, limited mobility, cognitive impairment, or poor access to cooling. During heat alerts, these individuals could receive phone calls or home visits, reminders about hydration, advice on medication risks, and transport to cooling centers when needed. Families and neighborhood volunteers could also help check indoor temperature, improve ventilation, and make sure that older adults are not left alone during high-risk periodscenter (Patel et al., 2022; Rodrigues et al., 2024; Núñez-Rodríguez et al., 2025b). These actions reflect the preventive logic of “Zhi Wei Bing” because they aim to preserve physiological reserve before heat stress develops into dehydration, collapse, or hospitalization. In this way, “Zhi Wei Bing” can be translated into a climate-health strategy that interrupts the resilience cascade at an early stage, rather than treating its downstream consequences.

### 3.2 The confucian ideal of “Tian-Ren-He-Yi”

While “Zhi Wei Bing” addresses physical reserves, the Confucian ideal of “Tian-Ren-He-Yi” (天人合一) provides the psychosocial stabilization required to complete the resilience framework. Rapid environmental degradation disrupts not only ecological balance but also the psychological well-being and autonomic stability of older adults. The sheer scale of the climate crisis frequently induces climate anxiety, existential distress, and a sense of “learned helplessness,” which are known to accelerate systemic inflammation and cognitive decline (Weimann and Opaliński, 2024). “Tian-Ren-He-Yi” offers a way to understand this problem by placing human life within a wider natural order. It does not mean that cultural belief directly changes physiological biomarkers. Rather, it may shape the social and psychological conditions, through which older adults experience and respond to climate stress. When environmental change is understood only as an uncontrollable external threat, older adults may experience fear, helplessness, and withdrawal (Wang et al., 2025). By contrast, a worldview that emphasizes balance between humans and nature may support a stronger sense of place, continuity, and shared responsibility. These psychological responses can matter for biological resilience. They may influence whether older adults seek help during extreme weather, whether families and communities organize care, whether individuals use green spaces for restoration, and whether climate risk is experienced as a shared challenge rather than an isolated personal burden. Through these pathways, Tian-Ren-He-Yi may indirectly support stress regulation, sleep quality, social connection, and recovery after environmental exposure (Ding et al., 2024). Its physiological relevance should therefore be understood as indirect and mediated through stress appraisal, behavior, social support, and environmental attachment.

In Confucian ethics, moral responsibility begins with human relationships but could extend to the natural worldtoward (Tang and Yu, 2023). Tian-Ren-He-Yi presents humans as part of a larger ecological order rather than as separate users of nature. This view differs from a narrow human-centered approach because it recognizes duties toward the environmentcheck (Ehrlich and Washington, 2013). It also differs from a purely nature-centered view because it still emphasizes human moral cultivation and responsible action. For older adults, this ethical orientation may support resilience by offering a sense of place, continuity, and responsibility when climate change disrupts familiar environments (Darmon, 2024). toward.

This environmental harmony is operationalized through two complementary Confucian virtues, Ren (仁) and Yi (义). These should be distinguished from ren (人, human) and yi (一, unity) in Tian-Ren-He-Yi (天人合一), where the phrase refers to the unity or harmony between humans and nature. Although these terms share similar Romanized forms, they refer to different Chinese characters and ethical meanings. Ren, often translated as benevolence, emphasizes care for others and attention to vulnerable people (Wang and Wang, 2025). In climate adaptation, Ren can support practical forms of care, such as checking on isolated older adults during heatwaves, sharing cooling resources, and building neighborhood networks. These Forms of social connections may reduce the risks of isolation by lowering chronic stress exposure and supporting healthier inflammatory and cardiovascular regulation (Gonzales et al., 2021; Appiah et al., 2022; Bhatia et al., 2023). Yi, often translated as righteousness or moral responsibility, emphasizes fairness and appropriate action. In the context of climate change, Yi may encourage communities to view older adults not only as recipients of protection, but also as participants in local adaptation. For example, older adults may contribute to neighborhood preparedness, community greening, or intergenerational climate education. Such participation may strengthen perceived agency, purpose in life, and intergenerational responsibility (Nygren et al., 2005; Syropoulos et al., 2025). These psychological resources do not replace physiological interventions, but they may help maintain motivation, social connection, and adaptive behavior under climate stress behavior. The historical roots of this integration can be seen in classical interpretations associated with Zheng Xuan, who emphasized the alignment of human conduct with seasonal and ecological rhythms (Sun and Zhang, 2025). This historical reference is used not as direct evidence of health effects, but as a cultural background for understanding why harmony, timing, and responsible conduct remain meaningful in behavior contemporary climate adaptation.

## 4 Policy and intervention implications

Modern climate policies often rely on measurable indicators such as carbon emissions, energy use, and household carbon footprints (Sayaka et al., 2024). Household carbon footprint studies show that income level and consumption capacity can strongly shape emissions, as higher income often increases demand for energy intensive goods and services (Du et al., 2024). This evidence is important because it shows that climate responsibility is unevenly distributed across social groups. However, an approach centered only on emissions accounting may overlook another form of inequality: some groups contribute differently to climate change but face much higher health risks from its consequences. Older adults may not always be the largest contributors to emissions, but they often face higher physiological risks from heatwaves, air pollution, and other climate hazards because of reduced resilience, chronic disease, limited mobility, and uneven access to cooling or care. For this reason, climate governance should connect mitigation with adaptation and care. Cultural ethics and traditional thoughts could help frame this connection in practical terms. Ren supports care for vulnerable groups, Yi emphasizes fair distribution of adaptation resources, and Tian-Ren-He-Yi links human health with ecological stability. Together, these principles suggest that climate governance should combine emission reduction with concrete protection for older adults, including heat warnings, cooling access, neighborhood care, and greener urban environments. The translation of these principles into contemporary health and climate governance is outlined in **Table 1**.

**Table 1:** Confucian ethical principles and their policy applications.

Confucian principle	Ethical meaning	Application in policy context	Real-world example
Ren (Benevolence)	Compassion and care for the vulnerable	Prioritizing adaptation for at-risk groups; equitable healthcare in climate response	Community heatwave protection networks targeting socially isolated older adults
Yi (Righteousness)	Fairness and moral responsibility	Fair burden-sharing and intergenerational justice; empowering older adults as climate actors	Engaging older adults in community greening and "silver economy" resilience projects
Tian–Ren–He–Yi	Harmony between humans and nature	Integrating ecological health into social policy; long-term sustainability	China's Ecological Civilization framework

To operationalize this Confucian-inspired approach and build systemic biological resilience, the following specific policy recommendations are proposed:

a. Prioritizing Vulnerable Communities through Interventions based on Ren: Climate adaptation policies must actively reduce suffering by supporting those most susceptible to physiological decline. From the perspective of Ren, climate-adaptive urban strategies can be understood as efforts to reduce avoidable suffering among vulnerable groups. In practice, this may include optimizing building layouts, providing cooling shelters, establishing urban ventilation corridors, creating shaded public spaces and expanding vertical greening to improve air circulation and mitigate urban heat island effects (Miralles-Guasch et al., 2019). When combined with neighborhood care networks, these environment and health integrations may help reduce heat exposure and physiological strain among older adults living alone during extreme heat events (Doherty et al., 2025). For example, measures such as heat warning systems, community outreach programs, home visits for socially isolated individuals, and access to cooling centers can support early risk identification and timely response (Adams et al., 2023; De Gea Grela et al., 2024; Oberai et al., 2025). At the same time, urban design strategies such as improved ventilation, shading, and green infrastructure can lower ambient temperatures and reduce cumulative heat exposure (Boni et al., 2024; Wang et al., 2025; Kang and Yoon, 2026). Together, these efforts may help ease cardiovascular stress, prevent dehydration, and reduce heat-related hospital visits among older adults.

b. Cultivating Yi (righteousness and fairness) via Equitable Resource Distribution and Resilience Literacy: Guided by righteousness and fairness, climate governance must ensure that adaptation resources are equitably distributed to protect fragile populations. Furthermore, rather than treating older adults merely as passive aid recipients, policies should cultivate “resilience literacy.” Resilience literacy refers to the practical ability to recognize climate-related health risks, understand warning messages, adopt protective behaviors, and use behavior available social and community resources (Haq and Gutman, 2021; Tipaldo et al., 2024). For older adults, this may include knowing the early signs of heat stress, adjusting outdoor activity during heat alerts, maintaining hydration, managing medications that increase heat vulnerability, and seeking timely help from family members, neighbors, clinics, or community workers. Resilience literacy should not be treated only as individual knowledge. It should also be supported by families, primary care services, and neighborhood organizations, especially for older adults with cognitive impairment, low income, or limited mobility. Providing them with the resources and platforms to participate in local sustainability efforts taps into their sense of intergenerational responsibility, thereby utilizing Yi to support perceived agency, social participation, and adaptive behavior under climate stress.

c. Implementing Tian-Ren-He-Yi for Long-term Ecological Stewardship: Climate strategies must shift away from short-term economic growth models to prioritize long-term ecological and public health. Recognizing the intrinsic value of nature aligns with the Confucian ideal of balance. China’s Ecological Civilization initiative serves as a prominent macro-level example. In pilot provinces for ecological civilization reform—namely Fujian, Jiangxi, and Guizhou—policy innovations span multiple institutional areas to build a harmonious relationship between humans and nature. By implementing ecological redlines, green space planning, and integrating public health objectives into urban design (as seen in cities like Guiyang and Fuzhou in China), these policies may generate population health co-benefits. Ecological redlines refer to areas designated for strict environmental protection to preserve key ecosystem functions, such as water regulation, biodiversity conservation, soil stability, and climate buffering. These protected spaces matter because they help maintain the environmental conditions that support health. Such interventions can improve air quality, mitigate urban heat island effects, and increase access to green and blue spaces that support both physical and psychological well-being (Becvarik et al., 2024). These measures can be understood as environment-mediated public health interventions, which do not function like clinical treatment. Instead, they improve the everyday conditions in which older adults live, move, rest, and recover (Bonham-Corcoran et al., 2022; Cosh et al., 2024). Cleaner air, lower heat exposure, shaded public space, and access to green and blue environments may reduce physiological stress and support psychological recovery among (Sealy Phelan et al., 2025). This is where the policy example connects with Tian-Ren-He-Yi. The concept does not simply ask people to appreciate nature in an abstract way. It asks human activity to remain within ecological limits (Zhuang and Liu, 2023). When urban planning protects ecological space, reduces heat, and improves living conditions for vulnerable groups, it gives institutional form to this idea of balance between humans and natural systems (Wang et al., 2026). In this way, ecological governance informed by traditional values can lead to tangible health benefits.

## 5 Conclusion

Climate change is rapidly emerging as a defining challenge for aging societies (Zhang et al., 2024a). Beyond its environmental consequences, climate stress increasingly disrupts the biological resilience of older adults by eroding physiological reserves and weakening the body’s capacity to recover from environmental shocks. As extreme heat, air pollution, and ecological instability intensify, the health of aging populations can no longer be understood solely through traditional disease-oriented models. Addressing this challenge therefore requires moving beyond fragmented biomedical perspectives toward a more integrative understanding of resilience (Prescott et al., 2022; Zhang, 2025).

Resilience must be conceptualized as a dynamic system shaped by the interaction between biological processes, psychosocial environments, and broader cultural frameworks. This perspective article has proposed an integrative framework for understanding resilient aging under climate change. This framework should be read as a hypothesis-generating perspective rather than as a claim that cultural ethics directly determine physiological biomarkers. Its main argument is that cultural values may shape the social and psychological conditions through which older adults respond to climate stress. At the physiological level, climate-related stressors can trigger a cascading disruption of biological resilience, in which repeated environmental exposures gradually weaken cardiovascular, immune, and neuro-regulatory systems. At the psychosocial level, social support, perceived agency, and community cohesion can buffer stress responses and facilitate recovery. At the cultural level, ethical traditions may serve as powerful normative and motivational forces that shape collective adaptation strategies and reinforce adaptive behaviors.

Within this integrative perspective, Confucian thought offers a valuable ethical lens. Principles such as harmony between humans and nature (Tian–Ren–He–Yi), benevolence (Ren), and righteousness (Yi) emphasize responsibility, empathy, and intergenerational care. These values encourage societies to recognize that environmental stewardship and the protection of vulnerable populations are inseparable goals. Rather than replacing modern scientific approaches to climate governance, such cultural insights complement them by embedding resilience strategies within broader moral and social commitments.

Importantly, integrating cultural ethics with contemporary climate governance resonates strongly with the emerging paradigm of planetary health, which emphasizes the inseparable relationship between human well-being and the stability of Earth’s natural systems (Ip, 2025). By bridging physiological science, social resilience, and ethical traditions, this approach provides a more comprehensive foundation for climate adaptation strategies that prioritize both health equity and ecological balance. Future research should therefore seek to operationalize the concept of biological resilience in aging populations through measurable indicators such as stress recovery dynamics, inflammatory regulation, and autonomic flexibility. Longitudinal studies linking climate exposure with aging trajectories are also needed to better understand how environmental stress accumulates across the life course. Interdisciplinary collaborations across gerontology, environmental science, public health, and cultural studies will be essential for developing culturally grounded resilience interventions for older adults.

Climate change presents not only a scientific and environmental challenge but also a societal test of how communities protect their most vulnerable members. Strengthening resilience among older adults requires integrating biological knowledge with social solidarity and ethical responsibility. By aligning cultural wisdom with modern planetary health strategies, societies may cultivate more inclusive and adaptive pathways toward healthy aging in an era of accelerating climate change.

## Data Availability

The original contributions presented in the study are included in the article/supplementary material, further inquiries can be directed to the corresponding author/s.
